# Microsurgical technique for femoral vascular access in the rat

**DOI:** 10.1016/j.mex.2017.11.005

**Published:** 2017-11-10

**Authors:** Michael George Zaki Ghali

**Affiliations:** Department of Neurobiology & Anatomy, Drexel University College of Medicine, 2900 Queen Lane, Philadelphia, PA, 19129, USA

**Keywords:** Catheterization, Vascular access, Femoral, Rat, Operative

## Abstract

Vascular access is used experimentally for a variety of reasons. In our lab, we achieve arterial access to record arterial pressure and venous access to administer fluids and drugs. We present a microsurgical atlas of our technique for femoral arterial and venous access in the rat.

## Graphical abstract

**Figure:**
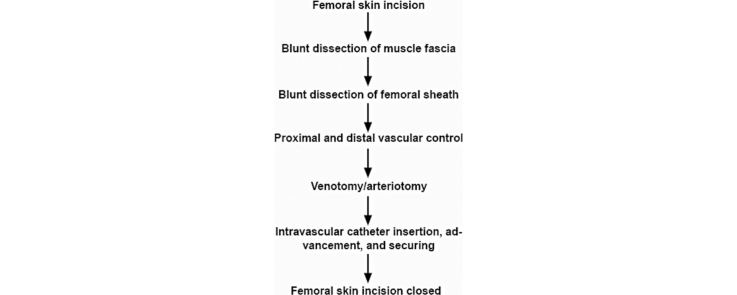

## Method details

Femoral arterial and venous access permits recording of arterial pressure and administration of fluids/drugs, respectively, in experimental animal models. In our lab, arterial pressure is used for general physiological monitoring, as well as a dependent variable during experimental interventions, including asphyxia, hypoxia, and hypercapnia, as well as procedures, including decerebration and spinal cord hemisection and transection [1], [2], [3], [4]. Venous access allows us to administer fluids and drugs, such as vecuronium, in unanesthetized decerebrate animals. Our technique is presented in descriptive and illustrative detail [1], [2], [3], [4], [5], [6].

## Methods

All procedures were approved by the Drexel University Institutional Animal Care and Use Committee, which oversees Drexel University’s AAALAC International-accredited animal program. Two catheter systems, one each for the femoral vein and artery, were prepared prior to experiments. The catheter system is composed of the catheter tubing itself − PE50 cut at a length of approximately 20 cm with the tip cut at a 45° angle with a razor blade. The PE50 tubing is secured over a needle attached to a three-way stopcock, with the two other ports attached to fluid-filled syringes. For the arterial system, one of these is a 1 ml syringe filled with 300 U/L of heparin in Ringer-Locke solution. The remaining are 5 ml syringes filled with normal saline, Ringer-Locke, or artificial CSF. The arterial catheter system is filled with heparin and the venous catheter system with non-heparinized solution. The stopcock is locked in the direction of the catheter tubing.

Ten spontaneously-breathing, Sprague-Dawley adult male rats (340–380 g) were anesthetized with isoflurane vaporized in O_2_ (Matrix; 4–5% induction, 1.85–2.15% maintenance) via a snout mask. The electrocardiogram (EKG) was measured via three small subcutaneous electrodes using conventional amplification and filtering (Neurolog; Digitimer, Hertfordshire, UK) and monitored using an audio amplifier (model AM10; Grass Instruments) and oscilloscope. Anesthetic depth was maintained at a level such that withdrawal reflexes and changes in heart rate in response to pinches of the distal hind limbs were absent.

**Fig. 1:**
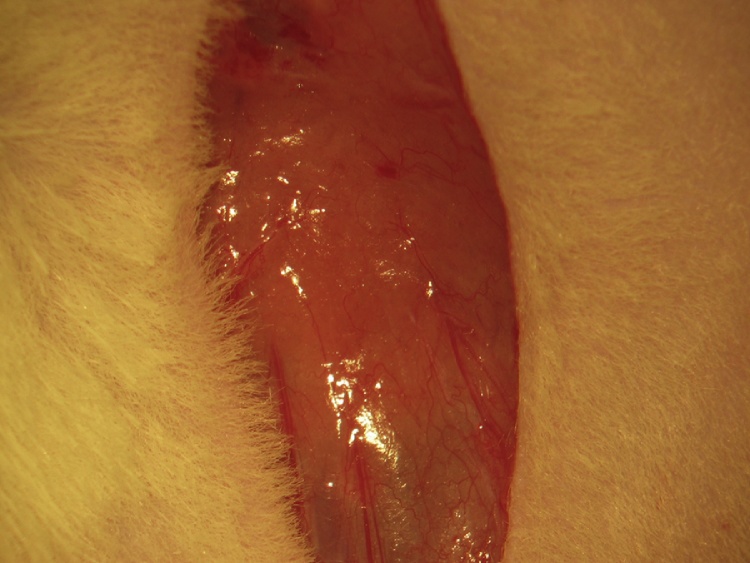
A femoral incision is made parallel to the femoral sheath.

**Fig. 2:**
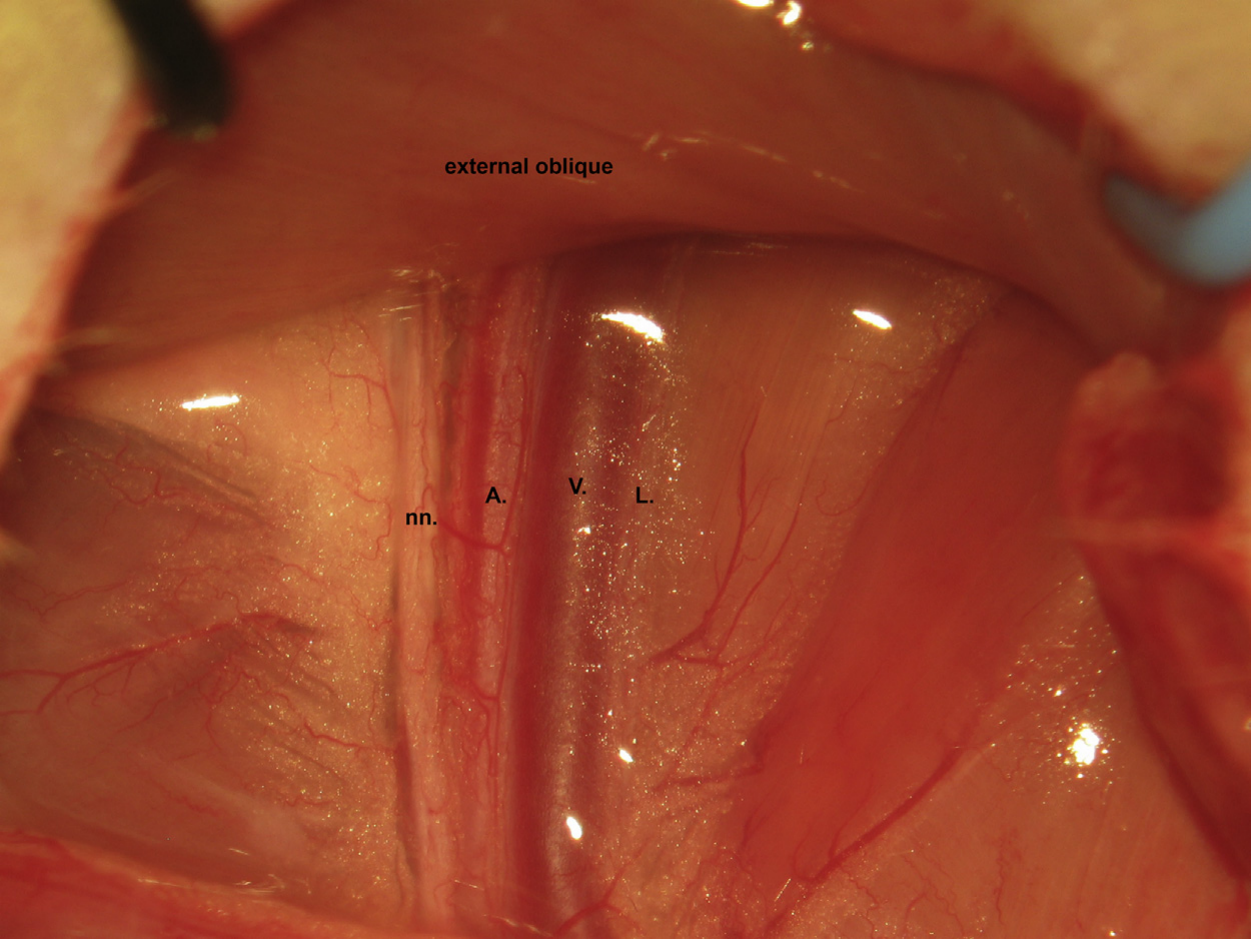
Soft tissue is dissected to expose the femoral neurovascular bundle. nn. nerves; A., artery; V., vein; L., lymphatics.

**Fig. 3:**
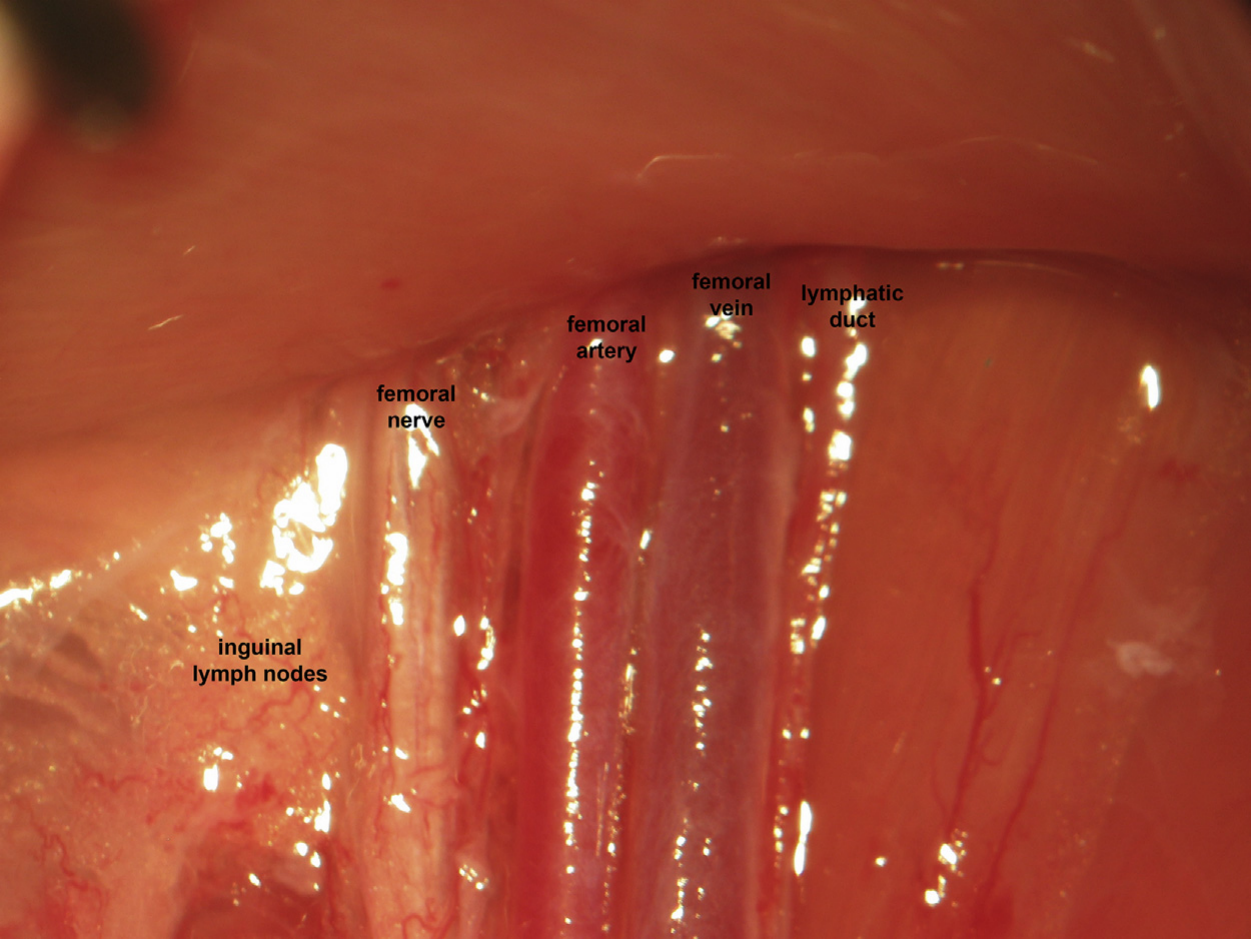
Magnified view of the femoral neurovascular bundle. nn. nerves; A., artery; V., vein; L., lymphatics.

**Fig. 4:**
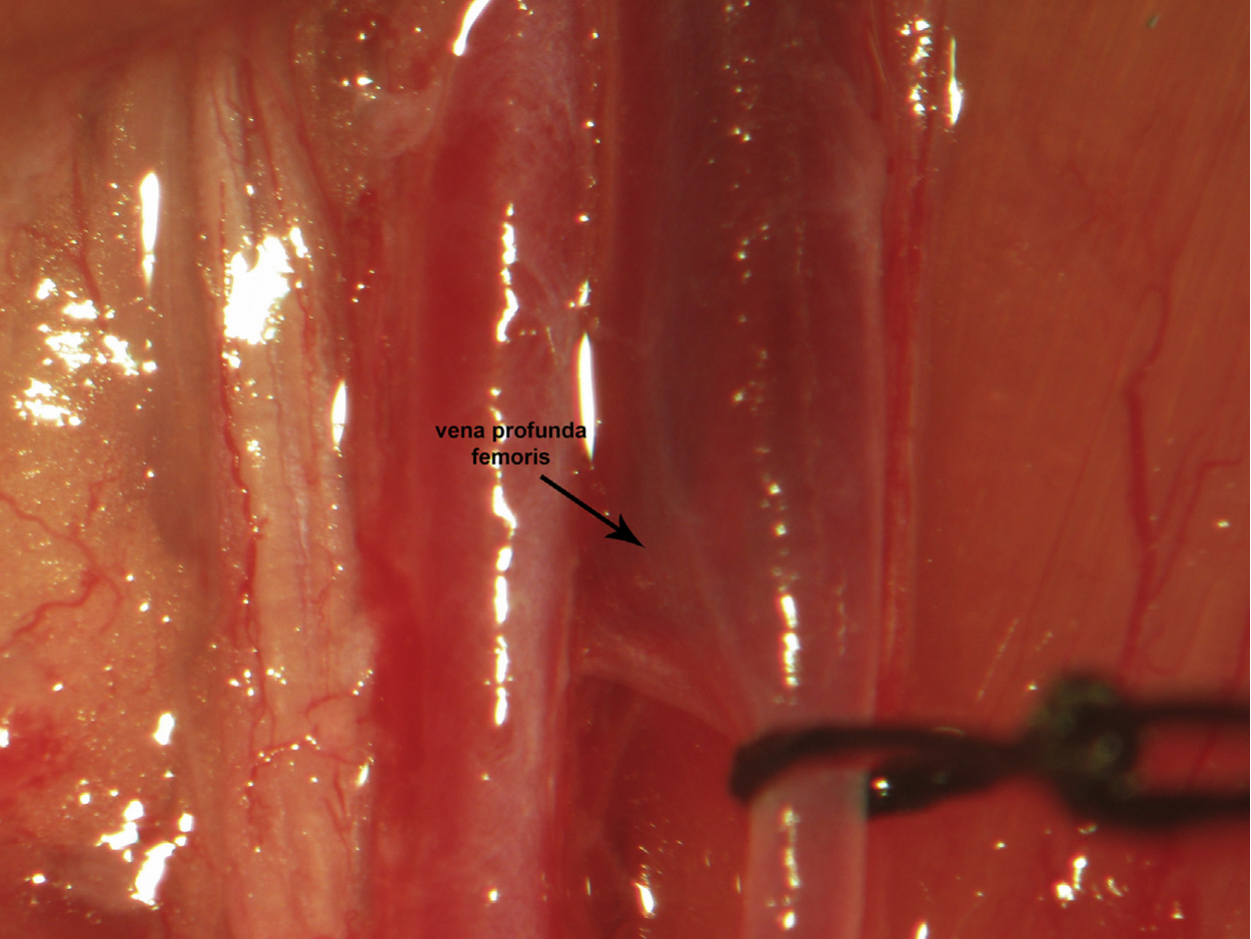
Medial retraction of the femoral vein reveals the vena profunda femoris. Proximal ligation should be distal to this branch to prevent bleeding through the venotomy site.

A skin incision is made in the femoral region parallel to the femoral sheath (Fig. 1). Soft tissue is dissected to expose the femoral neurovascular bundle (Fig. 2, Fig. 3). The femoral sheath is opened using blunt dissection directed along its long axis between the femoral artery laterally and vein medially. This exposes the femoral vessels, which are further separated with blunt dissection in the same manner. Medial retraction of the femoral vein reveals the vena profunda femoris (Fig. 4); proximal control should be distal to this branch to avoid retrograde bleeding via this vessel through the subsequent venotomy site. Distal ligation is obtained using 4-0 braided silk suture and a needle holder (or hemostat) is placed on the suture thread ends, approximately 3 cm from the knot and allowed to rest on the edge of the operating table. The operating table sits on the lab bench and the needle holder rests on the operating table and is supported by the lab bench, forming an approximately 30° angle with the horizontal plane. The weight of the needle holder applies an ideal degree of tension to the vessel to facilitate introduction of the catheter subsequently. This is a subtle yet important point for facile performance of the procedure, especially for insertion of the arterial catheter.

**Fig. 5:**
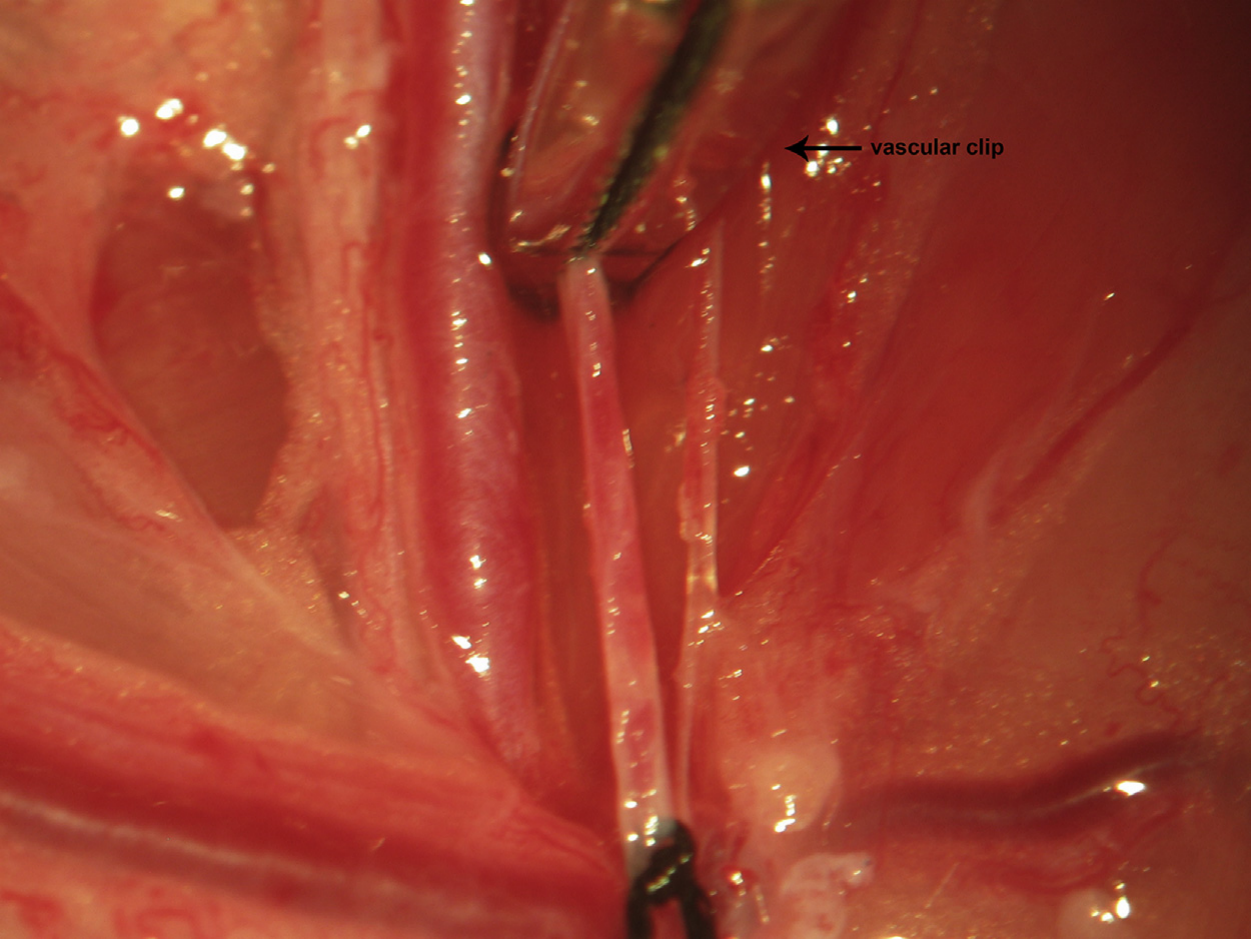
Following distal ligation using 4-0 braided silk suture, tension is applied to the suture threads using a needle holder (or hemostat), which is allowed to gently rest on the edge of the operating table (a custom-made block sitting atop the lab bench) and a temporary vascular clip is then applied distal to the vena profunda femoris to achieve proximal control.

**Fig. 6:**
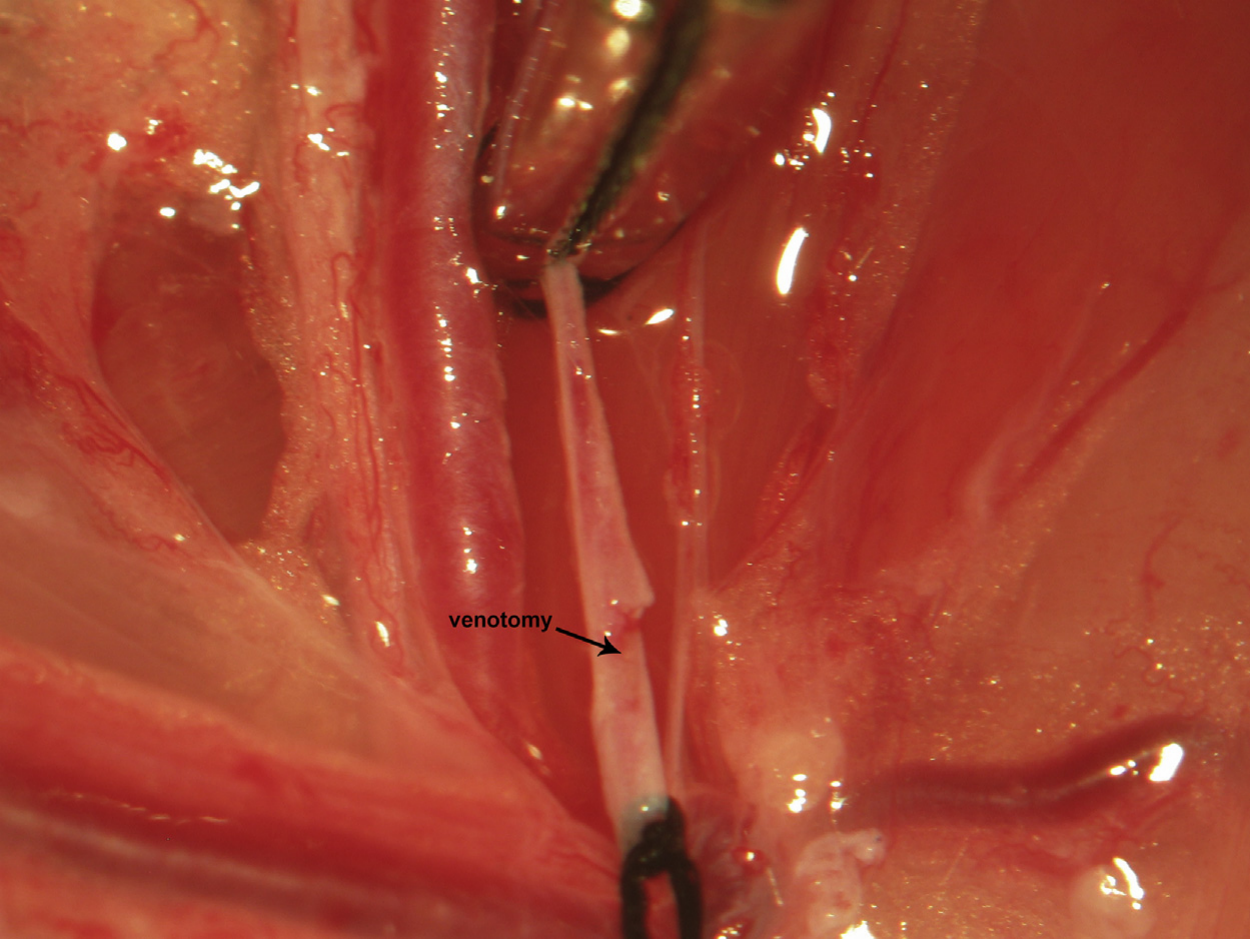
Proximal to the distal ligation site, a cut is made through the upper half of the vein’s circumference using iridectomy scissors oriented at a 45° angle.

**Fig. 7:**
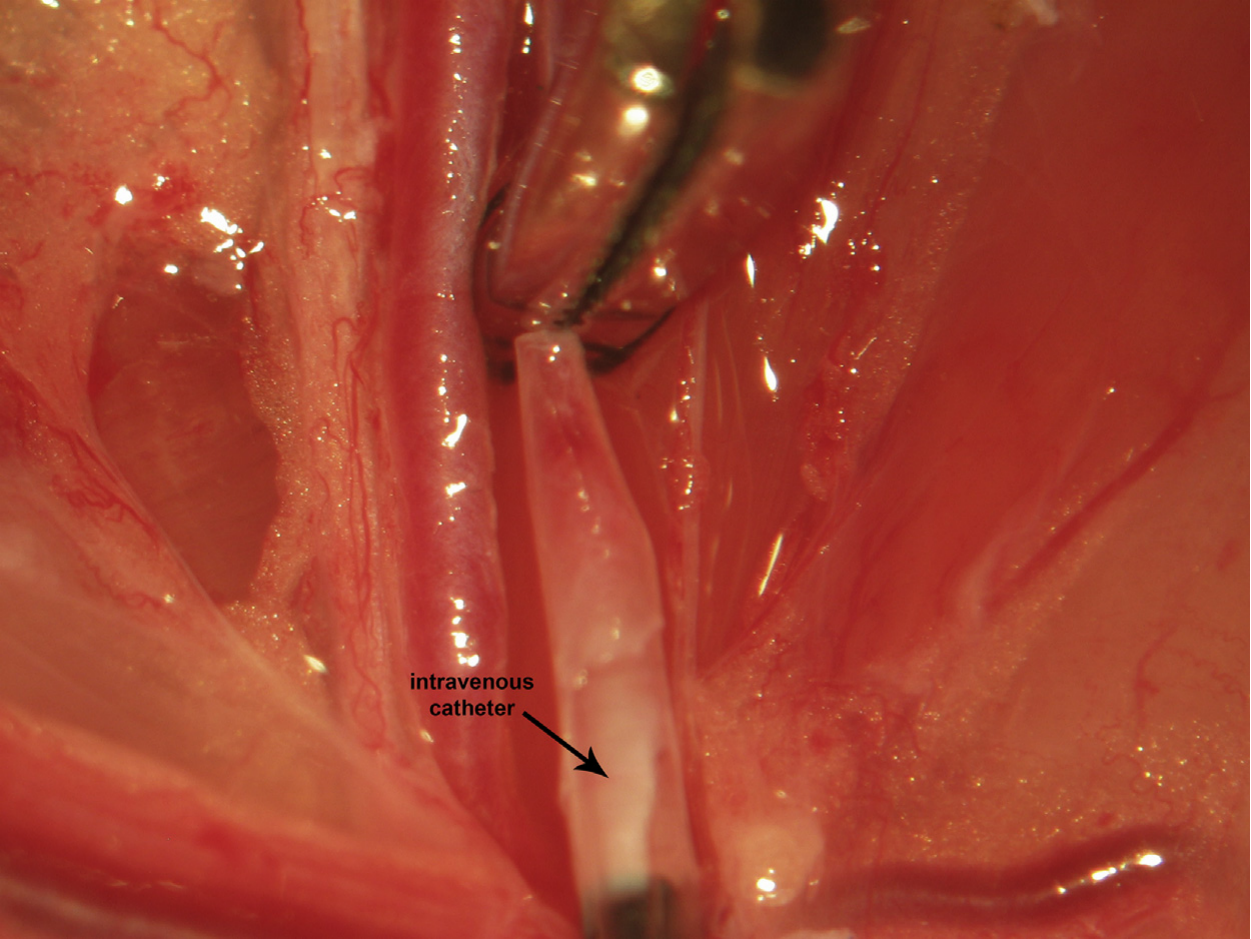
During catheter insertion, the system should be unlocked in direction of the catheter tubing and one of the attached syringes depressed to produce a fluid bubble at the catheter's tip in order to prevent introduction of air emboli to the vascular system. The catheter is inserted through the venotomy and advanced to the vascular clip. At this point, the catheter system is locked.

**Fig. 8:**
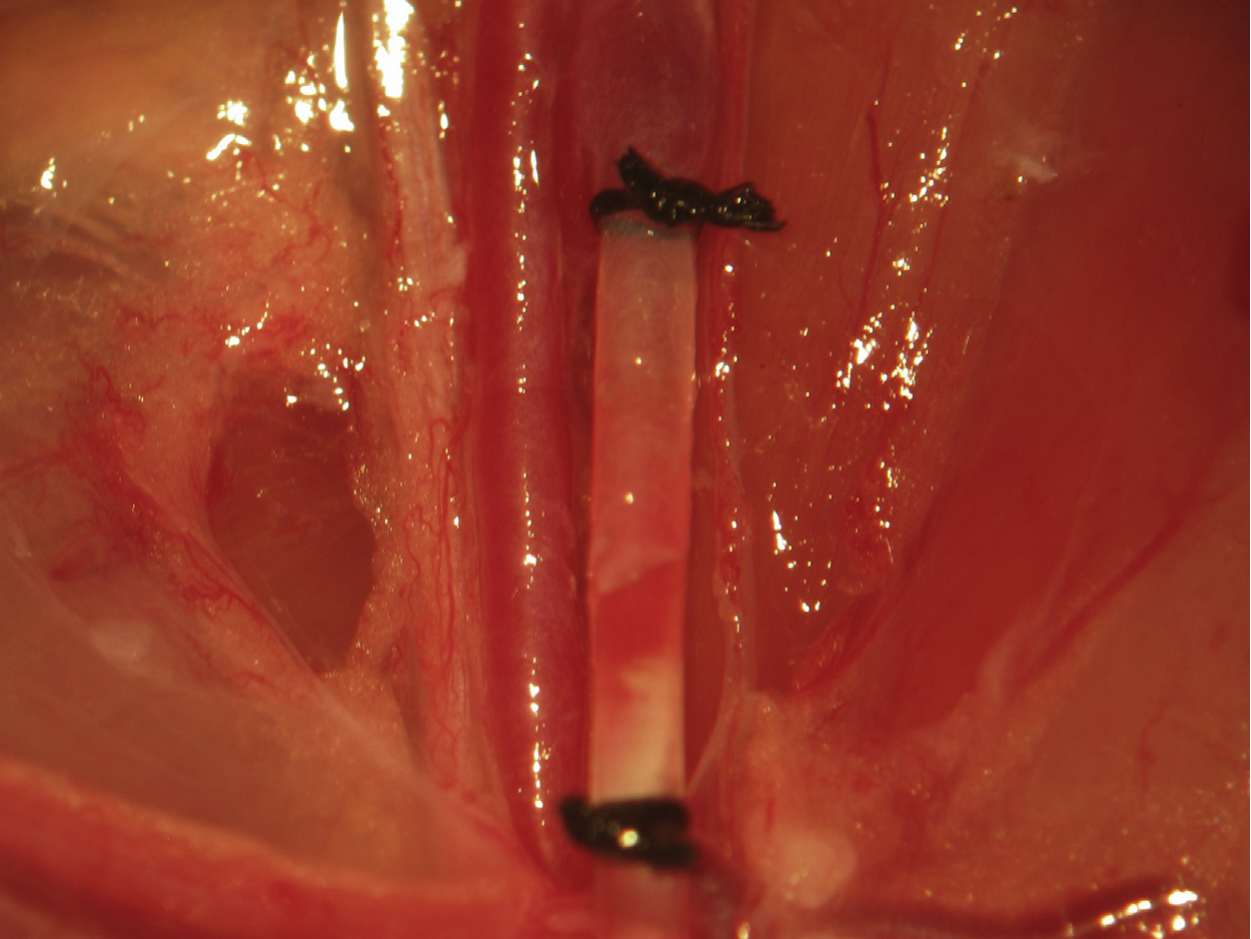
The vascular clip is removed and the catheter advanced distally to the level of the inguinal ligament. The catheter is secured to the femoral vein proximally and distally with 3 single knots using 4-0 braided silk suture.

Proximal control is then obtained via a vascular clip applied immediately distal to the vena profunda femoris (Fig. 5). One to two millimeters proximal to the distal ligation site, a venotomy is made through the upper half of the vessel circumference at a 45° angle using iridectomy scissors (Fig. 6). During catheter insertion, the stopcock is opened in the direction of the catheter proper and a fluid bubble should be present at the catheter's tip in order to prevent introduction of air emboli to the vascular system. The catheter is inserted through the venotomy and advanced to the vascular clip (Fig. 7). At this point, the stopcock is locked in the direction of the catheter. The proximal control vascular clip is then removed and the catheter advanced distally towards the level of the inguinal ligament (Fig. 8). The catheter is secured to the femoral vein proximally and distally with 3 single knots using 4-0 braided silk suture (Fig. 8).

If proximal and distal control are applied in the aforementioned manner, bleeding is not observed during venotomy or catheter insertion, but there is infrequently very slow oozing when advancing following removal of the proximal vascular clip. If observed earlier, it may be due to the proximal vascular clip being placed proximal to the vena profundal femoris. Rapid securing of the catheter to the vessel is necessary should this occur. Confirmation of catheter patency is achieved by unlocking the catheter, withdrawing ∼0.2 ml of venous blood then flushing with ∼1 ml.

**Fig. 9:**
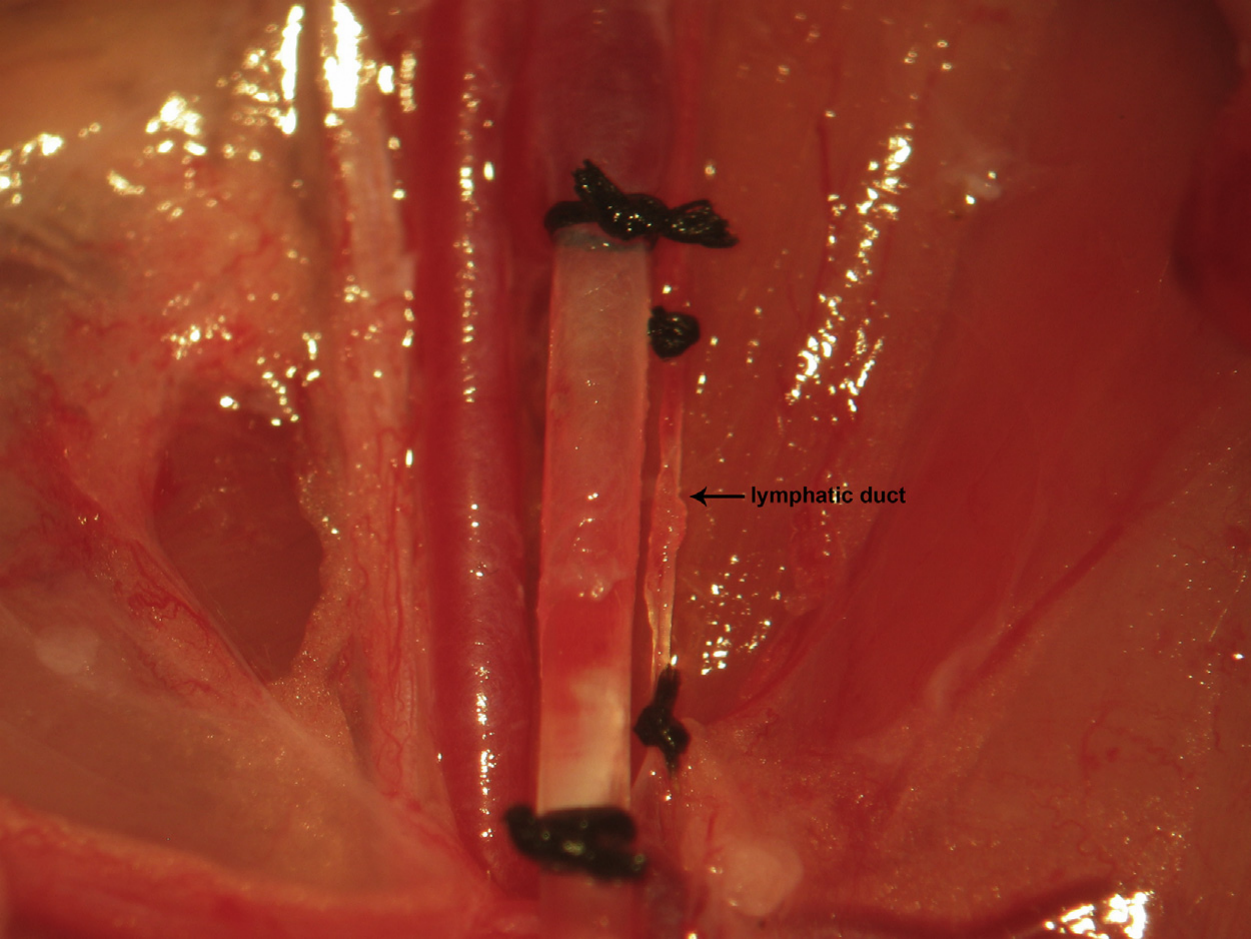
To prevent absorption of lactic acid and tissue breakdown products consequent to the ensuing ischemia resulting from femoral vascular catheterization, the lymphatics can be separately excluded from the circulation (shown) or alternatively included in the knots used to secure the femoral venous catheter.

**Fig. 10:**
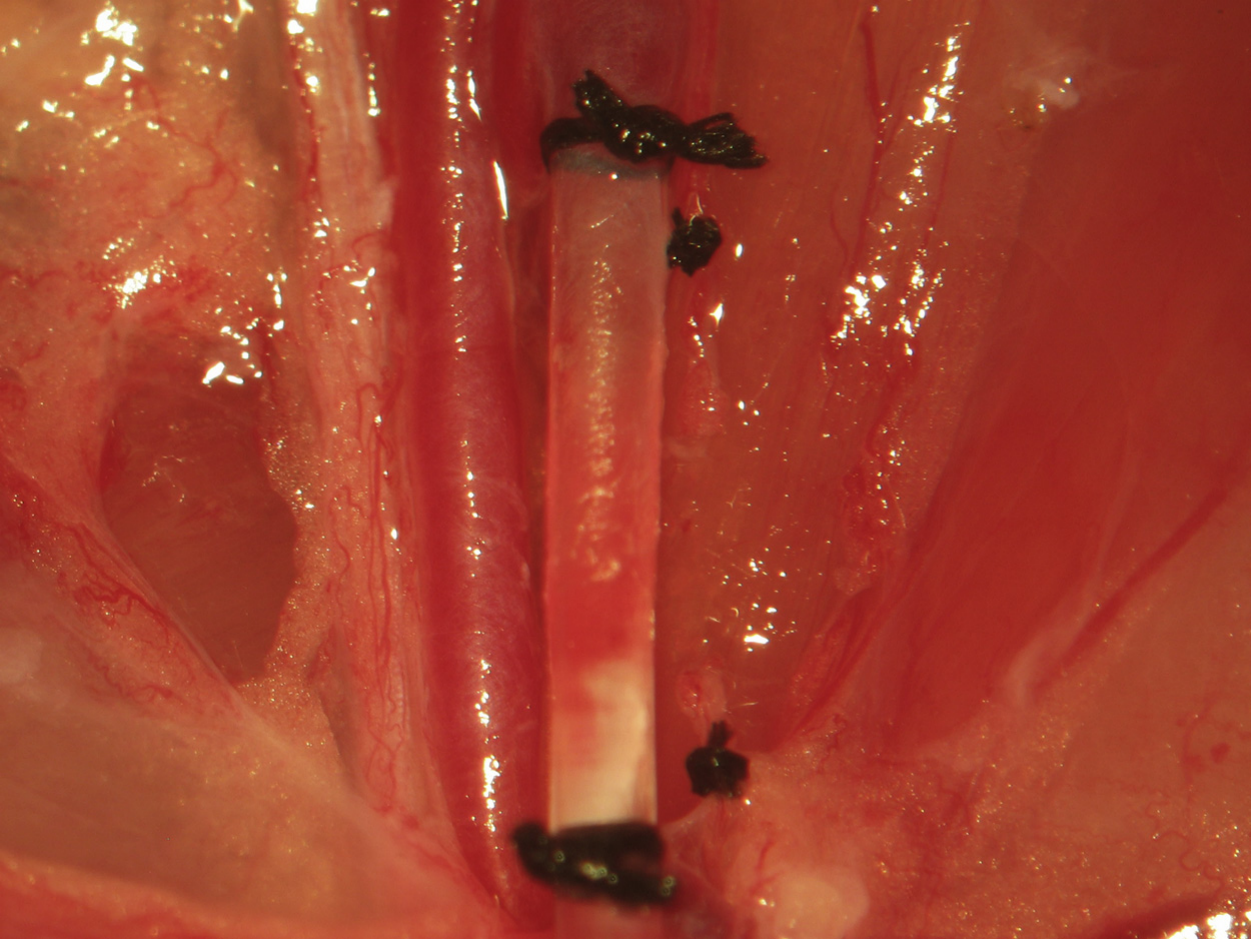
The lympathic duct is ligated proximally and distally and transected with iridectomy scissors. The field is then inspected for lymphatic leakage.

In order to prevent absorption of lactic acid and tissue breakdown products consequent to the ensuing ischemia resulting from femoral arterial catheterization, the lymphatics can be separately excluded from the circulation (shown) or alternatively included in the knots used to secure the femoral venous catheter (Fig. 9). The lymphatic duct is ligated proximally and distally, transected with iridectomy scissors, and the field is inspected for lymphatic leakage proximally and distally (Fig. 10). In our experience, lymphatic leakage is never observed. Should it occur, the proximal and distal ends should be sought and ligated individually, especially if neural recordings are to be performed in this region.

**Fig. 11:**
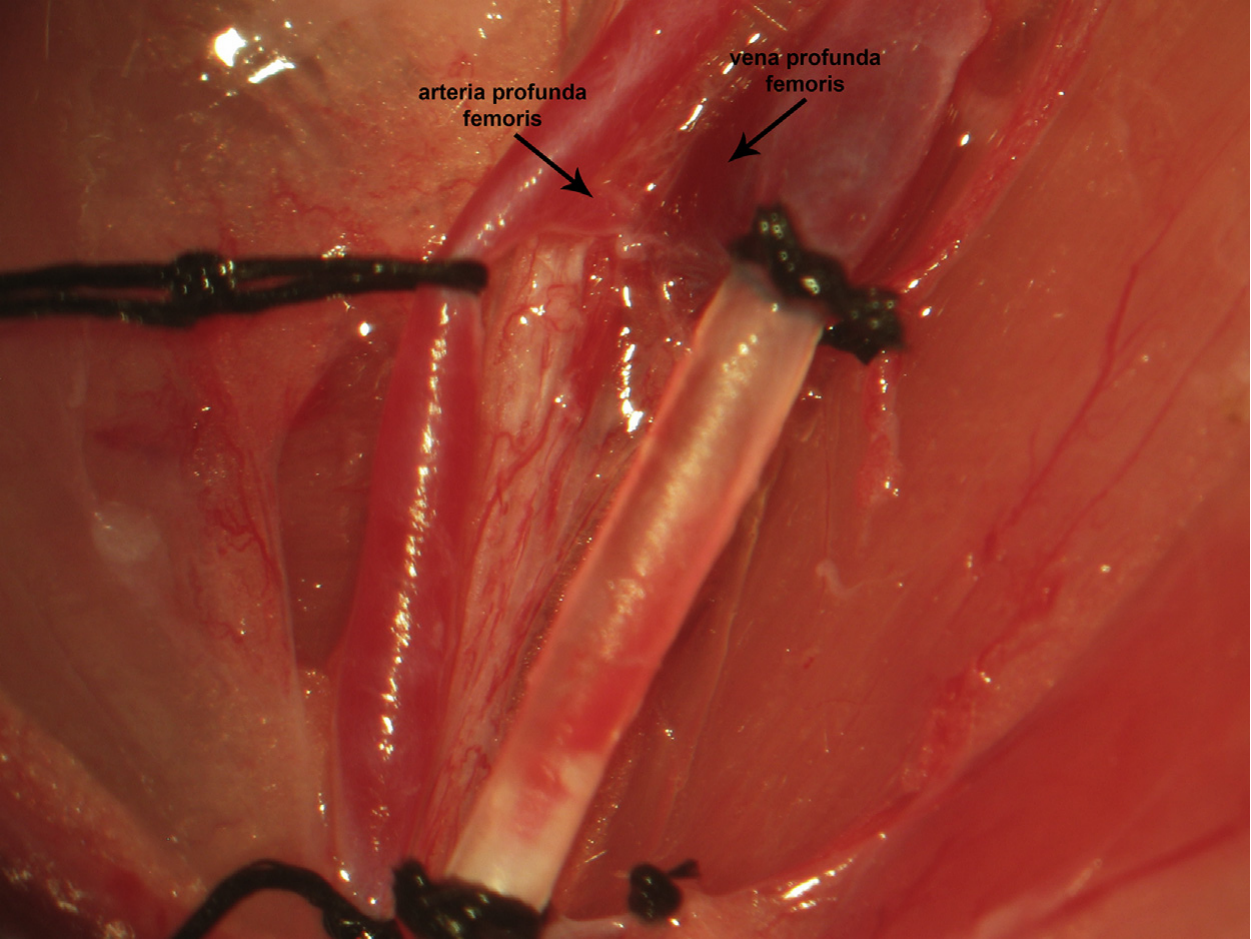
Lateral retraction of the femoral artery exposes the arteria profunda femoris, adjacent to the vena profunda femoris.

**Fig. 12:**
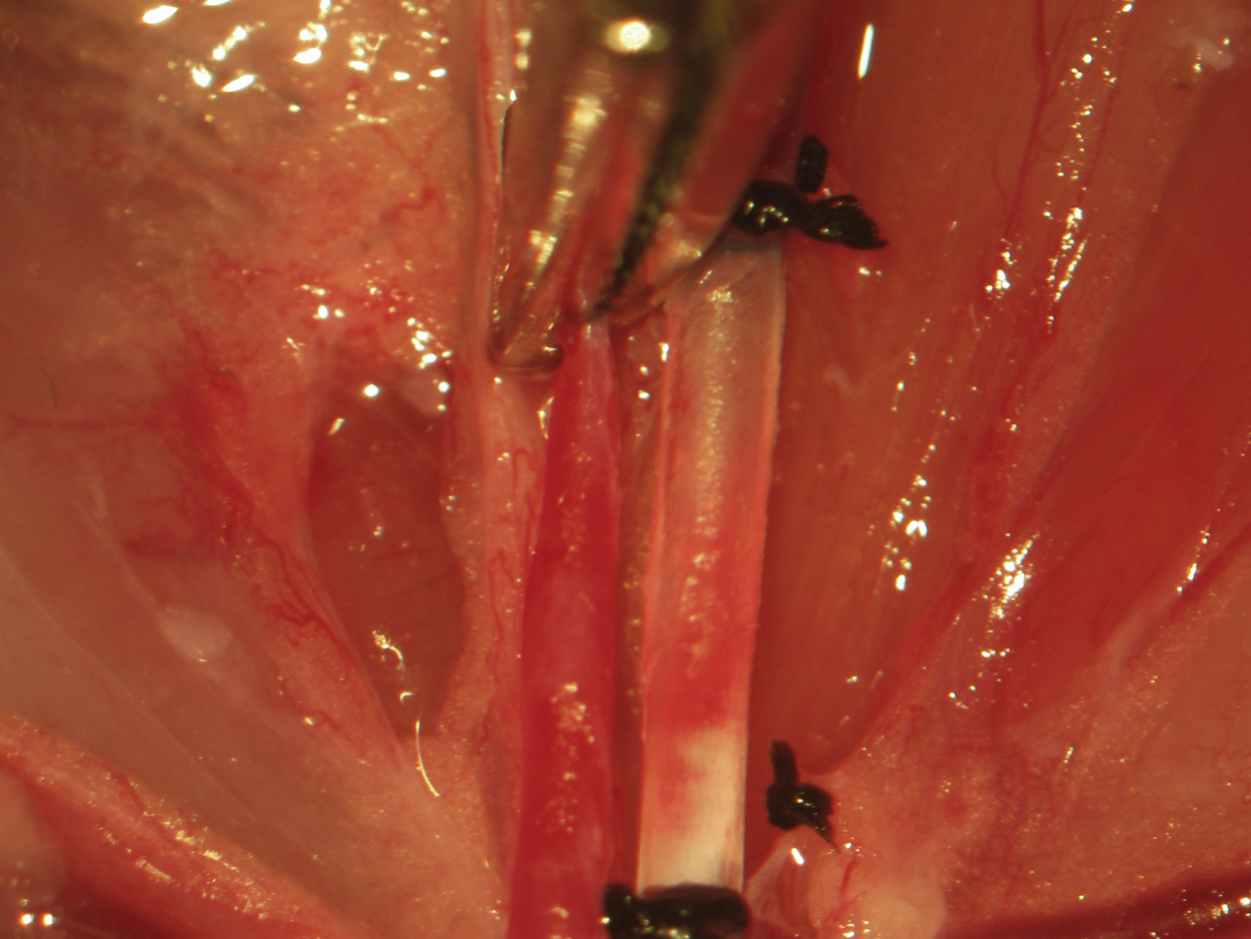
Following distal ligation, tension is applied to the suture used for the same and a vascular clip is applied distal to the arteria profunda femoris to achieve temporary proximal vessel occlusion.

**Fig. 13:**
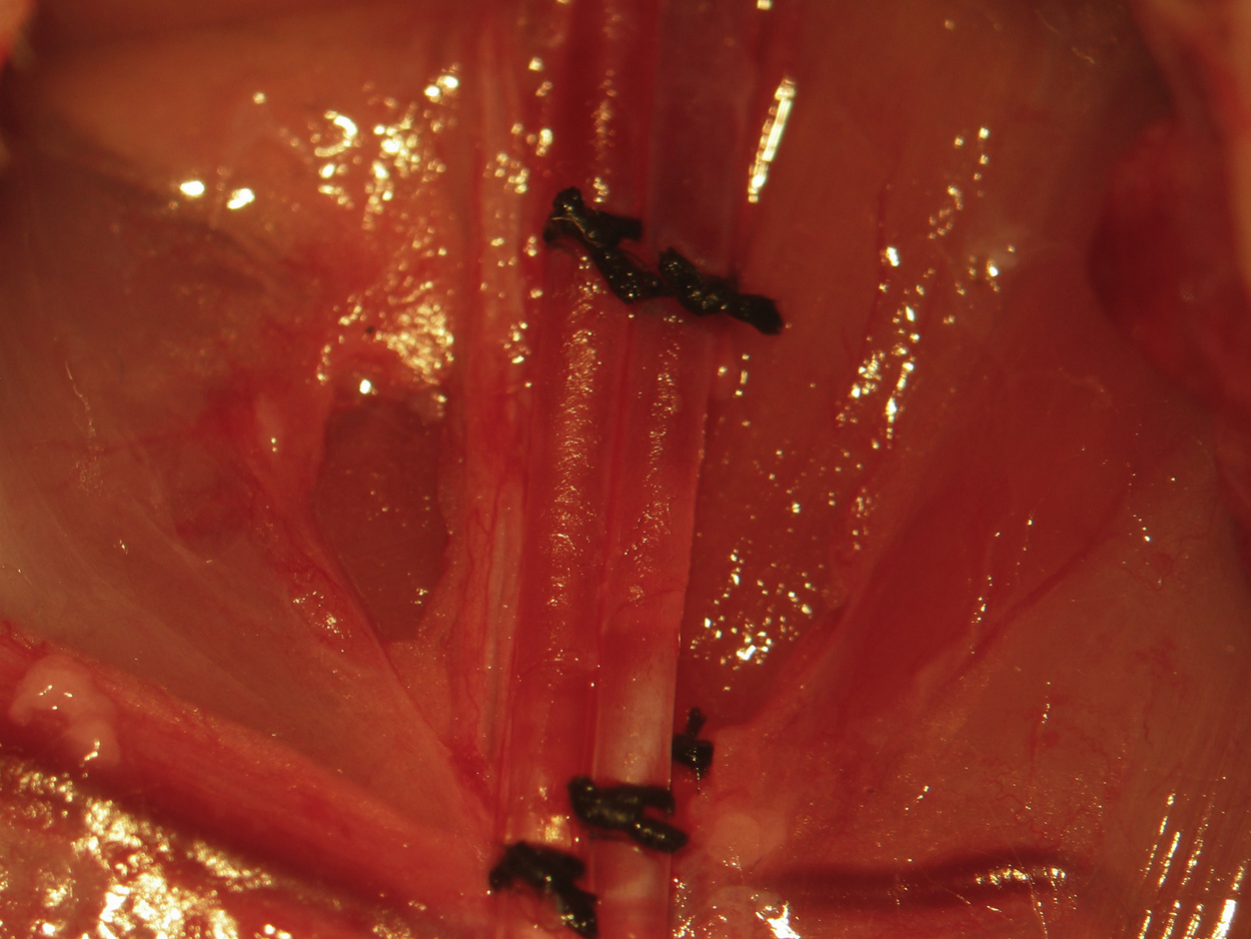
An arteriotomy is made immediately proximal to the distal ligation and a catheter (open) filled with heparin (300 U/L) in Ringer-Locke inserted and advanced to the vascular clip. The catheter is closed, the vascular clip is removed, and the catheter is further advanced towards the inguinal ligament. The catheter is secured proximally and distally using 3 single knots.

Femoral arterial access is obtained following femoral venous access. Lateral retraction of the femoral artery exposes the arteria profunda femoris, immediately adjacent to the vena profunda femoris (Fig. 11). Following distal ligation, tension is applied to the suture thread ends in the same manner as described previously for the femoral vein. Proximal vascular control is achieved by applying a temporary vascular clip immediately distal to the arteria profunda femoris (Fig. 12). An arteriotomy is made 1–2 mm proximal to the distal ligation and the catheter (open) is inserted and advanced to the vascular clip. Greater resistance is met when inserting the catheter intra-arterially than intravenously and the initial insertion will only permit the tip. The intra-arterial catheter is then advanced to the proximal clip by pulling the arterial wall over the catheter, having both secured with surgical instruments; this is distinct from venous catheter insertion, which is introduced quite easily. The stopcock is locked in the direction of the catheter, the proximal control vascular clip is removed, and the catheter is further advanced to the inguinal ligament. If proximal and distal control are applied in the aforementioned manner, bleeding is never observed during arteriotomy, catheter insertion, or advancement. The catheter is secured proximally and distally using 3 single knots (Fig. 13).

**Fig. 14:**
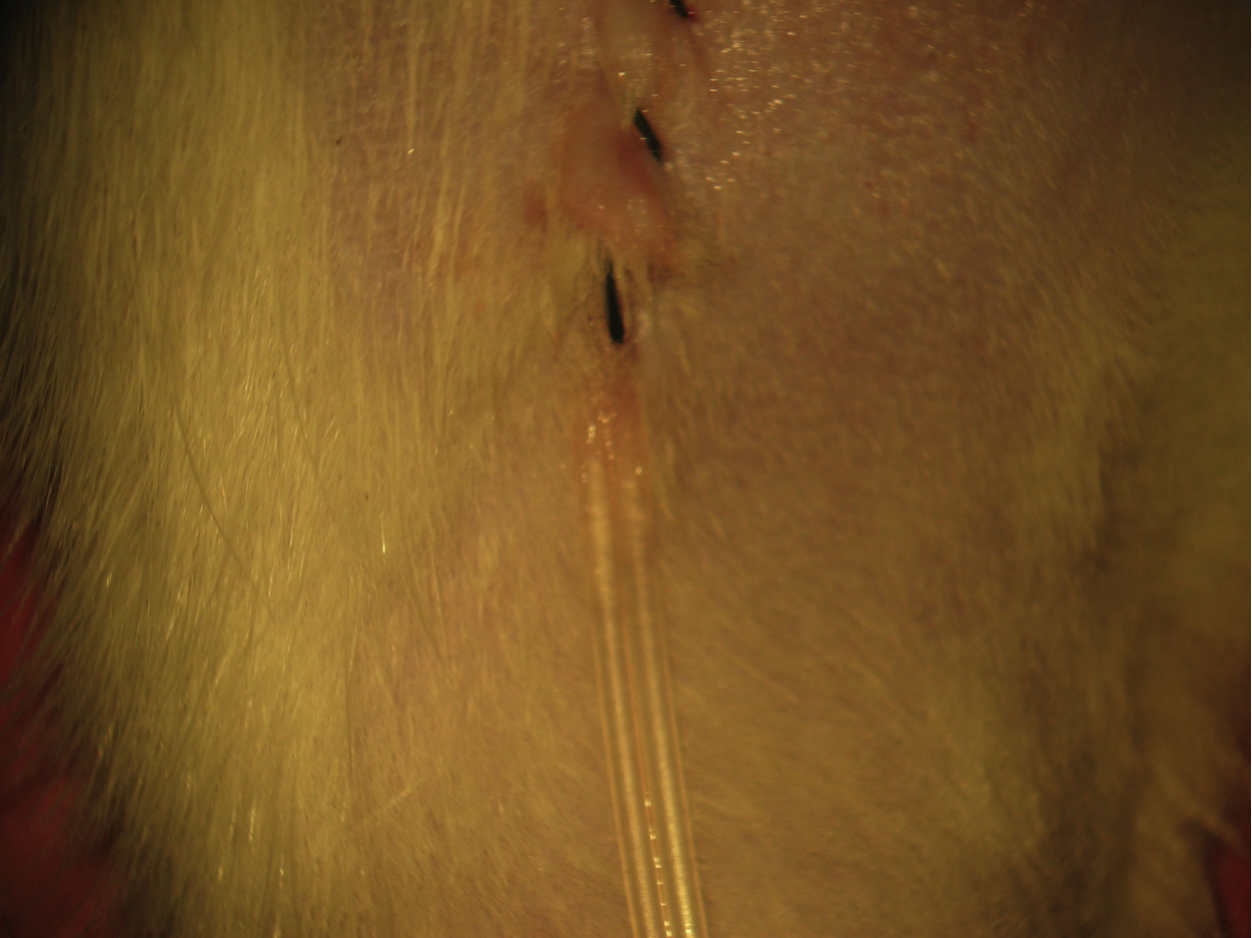
A Ringer-Locke soaked cottonoid is placed in the femoral region to replace removed soft tissue and the incision is closed continuously using 4-0 braided silk suture.

Confirmation of catheter patency is assured by observing arterial pulsations at the interface between the artery and distal catheter tip when the catheter is unlocked. As an option, a small amount of blood may be withdrawn and a similar amount flushed, but this may introduce a small amount of heparin into the animal, which could result in mild factor coagulopathy complicating further surgical preparation. We are typically sufficiently reassured of arterial catheter patency by visualizing normal arterial pulsations when the catheter is unlocked. A Ringer-Locke soaked cottonoid is placed in the femoral region to replace removed soft tissue bulk and the incision is closed using continuous 4-0 braided silk suture to prevent fluid loss to the environment and leakage during intraoperative changes in animal position for further pre-experimental microsurgical preparation (Fig. 14).

## Conclusion

We present our microsurgical technique for femoral venous and arterial access in the rat. It is important to identify the deep branches of the femoral artery and vein in order to optimally place proximal vascular control. It is important to introduce the catheter with a fluid bubble at the tip in order to prevent the introduction of air emboli and to ensure a continuous column of blood, with the latter of critical importance for obtaining an accurate recording of the arterial pressure waveform.

## Funding

Drexel University College of Medicine.
